# Risk for latent and active tuberculosis in Germany

**DOI:** 10.1007/s15010-016-0963-2

**Published:** 2016-11-19

**Authors:** Christian Herzmann, Giovanni Sotgiu, Oswald Bellinger, Roland Diel, Silke Gerdes, Udo Goetsch, Helga Heykes-Uden, Tom Schaberg, Christoph Lange, For the TB or not TB consortium

**Affiliations:** 1 Division of Clinical Infectious Diseases, Research Center Borstel, Borstel, Germany; 2 Center for Clinical Studies, Research Center Borstel, Borstel, Germany; 3 Epidemiology and Medical Statistics Unit, Department of Biomedical Sciences, University of Sassari, Sassari, Italy; 4 DAHW German Leprosy and Tuberculosis Relief Association, Würzburg, Germany; 5 Institute of Epidemiology, University Medical Hospital Schleswig–Holstein, Campus Kiel, Germany; 6 LungenClinic Grosshansdorf, Airway Research Center North, Großhansdorf, Germany; 7 Municipal Health Authority Hannover, Hanover, Germany; 8 Municipal Health Authority Frankfurt, Frankfurt, Germany; 9 Center of Pneumology, Agaplesion Deaconess Hospital Rotenburg, Rotenburg, Germany; 10 German Center for Infection Research (DZIF), Clinical Tuberculosis Unit, Borstel, Germany; 11 International Health/Infectious Diseases, University of Lübeck, Lübeck, Germany; 12 Department of Medicine, Karolinska Institute, Stockholm, Sweden

**Keywords:** LTBI, Incidence, Diabetes mellitus, IGRA, Health care workers, Household contacts

## Abstract

**Purpose:**

Few individuals that are latently infected with *M. tuberculosis* latent tuberculosis infection(LTBI) progress to active disease. We investigated risk factors for LTBI and active pulmonary tuberculosis (PTB) in Germany.

**Methods:**

Healthy household contacts (HHCs), health care workers (HCWs) exposed to *M. tuberculosis* and PTB patients were recruited at 18 German centres. Interferon-γ release assay (IGRA) testing was performed. LTBI risk factors were evaluated by comparing IGRA-positive with IGRA-negative contacts. Risk factors for tuberculosis were evaluated by comparing PTB patients with HHCs.

**Results:**

From 2008–2014, 603 HHCs, 295 HCWs and 856 PTBs were recruited. LTBI was found in 34.5% of HHCs and in 38.9% of HCWs. In HCWs, care for coughing patients (*p* = 0.02) and longstanding nursing occupation (*p* = 0.04) were associated with LTBI. In HHCs, predictors for LTBI were a diseased partner (odds ratio 4.39), sexual contact to a diseased partner and substance dependency (all *p* < 0.001). PTB was associated with male sex, low body weight (*p* < 0.0001), alcoholism (15.0 vs 5.9%; *p* < 0.0001), glucocorticoid therapy (7.2 vs 2.0%; *p* = 0.004) and diabetes (7.8 vs. 4.0%; *p* = 0.04). No contact developed active tuberculosis within 2 years follow-up.

**Conclusions:**

Positive IGRA responses are frequent among exposed HHCs and HCWs in Germany and are poor predictors for the development of active tuberculosis.

**Electronic supplementary material:**

The online version of this article (doi:10.1007/s15010-016-0963-2) contains supplementary material, which is available to authorized users.

## Introduction

Tuberculosis incidence has declined in Western Europe [1]. In 2014, the notified incidence in the European Union and European Economic Area (EU/EEA) was 14.2 cases per 100,000 population [1], in some countries only 5 cases per 100,000 [1]. Aiming at further reduction, the WHO advocated a target tuberculosis incidence of <1 cases per 1,000,000 in low incidence countries, *i.e.* the consensual tuberculosis elimination threshold [2].

Tuberculosis prevention relies on early case finding and on the identification of persons latently infected with *Mycobacterium tuberculosis* (LTBI) [3]. LTBI is defined by a positive response to the tuberculin skin test (TST) or an interferon-release assay (IGRA) without tuberculosis associated symptoms or signs. It is unclear whether the test results reflect viable bacilli in the human host. False positive results can be found, especially in populations of low prevalence.

Approximately 9% of healthy persons in Western Europe have a positive TST or IGRA test result [4]. If the whole population was screened with subsequent preventive chemotherapy for all who tested positive, the number needed to treat to prevent one case would not be cost effective. LTBI screening and treatment is, therefore, only performed in populations with an a priori higher risk for the disease [5], e.g. house hold contacts.

Contact tracing identifies 16–44% of close household contacts of contagious patients with LTBI [6, 7]. Nevertheless, only a small fraction develops active tuberculosis despite the absence of preventive chemotherapy [8]. Predictive markers of progression to active tuberculosis are lacking. The number of latently infected contacts requiring preventive chemotherapy to prevent a single case of tuberculosis is >1:30 in Western Europe [8]. Adherence to recommendations for preventive chemotherapy is poor in Germany [9].

To improve prevention and to target individuals for preventive chemotherapy more precisely, additional knowledge of risk factors for of LTBI and tuberculosis is needed. Therefore, our consortium collected epidemiological and clinical data from pulmonary tuberculosis (PTB) patients and close contacts.

## Methods

This observational, multicentre, prospective study was conducted by the German research consortium on “pulmonary tuberculosis—host and pathogen determinants of resistance and disease progression—(TB or not TB)”. It was approved by the ethics committee of the University of Lübeck (reference 07–125) and adopted by the ethics committees of all 18 participating centres.

Household contacts (HHCs) were recruited at three municipal healthcare centres (Frankfurt, Hamburg, Hannover). They were suitable for enrolment if they were asymptomatic with no signs of tuberculosis on chest X-ray, were exposed >8 h to patients with acid-fast bacilli (AFB) in the sputum or >40 h in AFB negative, culture-confirmed PTB. Their last unprotected exposure was ≥8 weeks prior to enrolment.

Healthcare workers (HCWs) with ongoing professional contact to patients with AFB sputum smear-positive tuberculosis, a cumulative exposure of ≥2 years, and no signs or symptoms of tuberculosis were recruited at 18 German respiratory medicine centres.

Patients with current or previous PTB that was culture confirmed from sputum or a broncho-pulmonary specimen were enrolled. Patients were excluded if they were prison inmates, subjects under guardianship or soldiers as this interferes with their informed consent.

Clinical and demographic data were captured on an ad hoc standardised questionnaire.

This questionnaire was amended during the first 3 years resulting in some data on medical and social risk factors. IGRAs were performed on blood from contact persons by the QuantiFERON Gold In-Tube^®^ (QFT; Cellestis Qiagen, Australia) or T-Spot.TB^®^ (Elispot; Oxford Immunotec, Oxford, UK) at the physician’s discretion. TST was not regularly performed since national recommendations regarding the use of the TST were amended during the study [10].

At the start of the study, a two-step approach was recommended involving both, TST and IGRA but TST was abandoned in 2011. The study protocol required an obligatory IGRA and an optional TST.

After 2 years, HHCs were followed up by health authorities; HCWs by a study physician. A questionnaire was sent to the participants. Notified tuberculosis cases would have been reported by the participating health authorities.

### Statistical analyses

Variables were described using relative frequencies (percentages) and means (standard deviations—SD) or medians (interquartile ranges—IQR) according to their parametric distribution, respectively.

Chi-squared test and student *t* test or Mann–Whitney *U* test were applied appropriately to assess significant differences. Appropriate univariate and multivariate logistic regression analyses were performed.

Two-tailed *p* values <0.05 were considered significant. Analyses were carried out with STATA^®^13 software (StataCorp, College Station, TX, USA).

## Results

From December 2008 to December 2014 we recruited 1754 individuals, i.e. 603 HHCs, 295 HCWs, and 856 PTB patients (Table 1). 81 HHCs and 15 HCWs were excluded from further analyses due to a missing IGRA result. Among contact persons (Table 2), HCWs were older than HHCs (mean ± SD: 48.0 ± 5.0- vs 40.6 ± 9.3 years; *p* < 0.0001), were less likely to smoke (23.5 vs 46.4%; *p* < 0.0001), more likely to be female (88.0 vs 53.6%; *p* < 0.0001), and to be born in Germany (88.4 vs 53.2%; *p* < 0.0001) by a German mother (85.9 vs 43.6%; *p* < 0.0001). An IGRA result was available in 522 HHCs and 280 HCWs, of which 180 (34.5%) and 109 (38.9%) had a positive result, respectively. IGRA results had no gender preference. The proportions of males were 47.8 vs 45.6% (*p* = 0.64) among HHCs and 21.3 vs 22.5% among HCWs (*p* = 0.82) with a positive vs. negative IGRA result, respectively. Most of the initial patients had a TST, but due to changed recommendations, later patients did not necessarily undergo skin testing. Policy changes were adapted by the centres with variable delay. For HCWs, neither a well-defined period of exposure nor the moment of last unprotected exposure could be determined. Preventive therapy is not recommended in this setting; therefore, no data about preventive chemotherapy was obtained. Most HHCs were recruited about 6–8 weeks after their last unprotected exposure, i.e. before isoniazid preventive therapy (IPT) was initiated. Therefore, data on IPT was only available in about one-third of the individuals. IPT was administered more often in IGRA-positive persons (11 out of 59 subjects; 18.6%) than IGRA-negative subjects (3 out of 109 subjects, 2.8%; *p* < 0.0001). No contact person developed active tuberculosis during the two-year follow-up. Follow-up data was available for 241 HCWs and 171 HHCs.

**Table 1:** Numbers of study subjects from participating centres

Municipal health authorities/centre	Number of HHCs	Number of PTBs
Hamburg municipal health authority	154	47
Frankfurt municpal health authority	271	132
Hannover municipal health authority	149	97
Research Center Borstel	29	See below
Total	603	276

Respiratory hospital	Number of HCWs	Number of PTBs
Lungenfachklinik immenhausen	16	31
HELIOS Klinikum, Berlin	40	5
Klinikum Nürnberg	19	28
Lungenklinik Hemer	8	0
Asklepios München-Gauting	26	245
Thoraxklinik UK Heidelberg	0	12
Fachkrankenhaus Coswig	8	41
Klinik Bad Lippspringe	0	0
Lungenklinik Lostau	21	0
Pneum.Klinik Greifenstein	30	20
Klinik Donaustauf	25	7
Klinikum Hannover	9	0
Fachklinik, Parsberg	16	0
Klinik Schillerhöhe Gerlingen	0	0
KKH Diekholzen	9	8
Klinik St. Blasien	1	0
Ev. Lungenklinik Berlin	6	3
Klinik Amsee, Waren	0	0
Research Center Borstel	11	87
Lungenklinik Rotenburg	19	5
Krankenhaus Großhansdorf	12	42
MH Hannover	8	0
KH Solingen	11	31
Dortmund	0	15
Total	280	580

*HHCs* household contacts, *HCWs* health-care workers, *PTBs* patients with pulmonary tuberculosis

**Table 2:** Risk factors for LTBI and active TB in household contacts, healthcare workers and patients with pulmonary tuberculosis

	HHCs				HCWs					PTBs	*p* value (all HHCs vs. all HCWs)	*p* value (all HHCs vs. all PTBs)	*p* value (all HCWs vs. all PTBs)
	All (*n* = 522)	IGRA− (*n* = 342)	IGRA+ (*n* = 180)	*p* value IGRA− vs IGRA+ HHCs	All (*n* = 280)	IGRA− (*n* = 171)		IGRA+ (*n* = 109)	*p* value IGRA− vs IGRA+ HCWs	all (*n* = 856)			
Male *n* (%)	242/522 (46.4)	156/342 (45.6)	86/180 (47.8)	0.64	61/277 (22.0)	38/169 (22.5)		23/108 (21.3)	0.82	568/856 (66.4)	<0.0001	<0.0001	<0.0001
Mean (SD) age, years	40.6 (15.0)	39.1 (14.8)	43.3 (15.0)	0.03	48.0 (9.3)	46.3(9.2)		50.6 (8.9)	<0.0001	47.1 (16.4)	<0.0001	<0.0001	0.38
Migration background													
Born in Germany *n* (%)	266/519 (53.2)	191/340 (56.2)	85/179 (47.5)	0.06	244/276 (88.4)	151/169 (89.4)		93/107 (86.9)	0.54	433/855 (50.6)	<0.0001	0.36	<0.0001
Domestic risk factors													
TB disease of the partner *n* (%)	61/200 (30.5)	31/130 (23.9)	30/70 (42.9)	0.005	5/270 (1.9)	3/163 (1.8)		2/107 (1.9)	1.0	58/689 (8.4)	<0.0001	<0.0001	0.0003
TB disease of a sibling *n* (%)	30/443 (6.8)	12/294 (4.1)	18/149 (12.1)	0.002	8/269 (3.0)	4/162 (2.5)		4/107 (3.7)	0.72	67/747 (9.0)	0.03	0.16	0.01
TB disease of a child *n* (%)	16/136 (11.8)	8/82 (9.8)	8/54 (14.8)	0.37	3/266 (1.1)	2/163 (1.2)		1/103 (1.0)	1.0	33/549 (6.0)	<0.0001	0.02	0.01
Environmental risk factors													
Tobacco smoking *n* (%)													
Current	232/500 (46.4)	147/326 (45.1)	85/174 (48.9)		61/260 (23.5)	38/165 (23.0)		23/95 (24.2)	0.83	57/116 (49.1)	<0.0001	0.59	<0.0001
Former	313/505 (62.0)	202/331 (61.0)	111/174 (63.8)		145/262 (55.3)	90/166 (54.2)		55/96 (57.3)	0.63	18/116 (15.2)	0.08	<0.0001	<0.0001
Medical risk factors													
Anti-TNF treatment *n* (%)	0/253 (0.0)	0/164 (0.0)	0/89 (0.0)	–	0/253 (0.0)	0/162 (0.0)		0/91 (0.0)	–	15/843 (1.8)	–	0.03	0.03
Alcohol dependency *n* (%)		3/164 (1.8)	12/89 (13.5)	<0.0001						127/846 (15.0)		<0.0001	
Diabetes mellitus *n* (%)	10/251 (4.0)	4/163 (2.5)	6/88 (6.8)	0.09	18/260 (6.9)	14/165 (8.5)		4/95 (4.2)	0.22	66/852 (7.8)	0.14	0.04	0.63
HIV-positivity *n* (%)	0/234 (0.0)	0/153 (0.0)	0/81 (0.0)	–	7/250 (2.8)	5/160 (3.1)		2/90 (2.2)	1.0	33/835 (4.0)	0.01	0.002	0.38
Intravenous drug usage *n* (%)	14/253 (5.5)	2/164 (1.2)	12/89 (13.5)	<0.0001	5/262 (1.9)	4/166 (2.4)		1/96 (1.0)	0.66	29/852 (3.4)	0.03	0.12	0.22
Previous mycobacterial exposure and diagnostics													
BCG vaccination *n* (%)	193/297 (65.0)	122/187 (65.2)	71/110 (64.6)	0.90	147/251 (58.6)		88/155 (56.8)	59/96 (61.5)	0.46	193/433 (44.6)	0.12	<0.0001	0.0004

*HHCs* household contacts, *HCWs* health-care workers, *SD* standard deviation; *BCG* bacillus calmette-guerin, *TB* tuberculosis, *IGRA* interferon-gamma release assay, *IGRA*+ positive IGRA result, *IGRA*− negative IGRA result, *QFT* quantiferon test, *PTBs* patients with pulmonary tuberculosis, *TST* tuberculin skin testing

While a univariate analysis suggested that the rate of IGRA positivity increased with age in the HHCs and HCWs, this was not confirmed in a multivariate logistic regression analysis (Odds ratio 0.99, 95% confidence interval 0.97–1.02; Supplementary material Table 3). IGRA positivity was independent of the person’s migration background. Among the HHCs, IGRA positivity correlated with the size of a simultaneous TST, while previous positive TSTs as reported by HHCs did not predict a positive IGRA. In the HCWs, IGRA positivity was more likely in persons who reported a positive previous TST (86.5%) than in subjects who reported a negative previous skin test (53.3%; *p* value <0.0001) (Table 2).

The strongest predictor for LTBI after domestic exposure (Supplementary material Table 3) was a partner with PTB (OR 4.39, 95% CI 1.88–10.26). The univariate analysis suggested that a diseased sibling was also associated with LTBI (OR 3.23, 95% CI 1.51–6.90), but this was not confirmed in the multivariate logistic regression analysis (OR 1.23; 95% CI 0.28–5.36). Sexual contact with the partner increased the prevalence of LTBI from 30.8% (no sex) to 53.3% (sexual activity). Other factors associated with a positive IGRA result among HHCs were a history of previous tuberculosis (4.5 vs 0.3%; *p* = 0.001), IVDU (13.5 vs 1.2%; *p* < 0.0001), and alcohol dependency (13.5 vs 1.8%; *p* = 0.0001).

For the HCWs, exposure to family members with PTB was not associated with LTBI, underlining the focus on professional exposure. Caring for coughing patients (*p* = 0.02) and a duration of >20 years of occupation (*p* = 0.004) were associated with LTBI, while the use of respirators, surgical masks, negative pressure rooms or white coats were not (Table 2).

Patients with PTB born in Germany (*n* = 433) were older than tuberculosis patients born abroad (*n* = 422) with a mean (±SD) age of 51.8 (±16.2) vs. 42.4 (±15.1) years at diagnosis (*p* < 0.0001). Uninfected and latently infected HHCs showed a balanced gender distribution but PTB was more prominent in men (*n* = 568, 66.4%). Foreign-born TB patients were more likely to live in an urban environment (90.7%) than Germany born patients (70.6%; *p* < 0.0001). Domestic exposure to a diseased partner or child increased the risk of LTBI. In patients with PTB, grandparents (9.1%), partners (8.4%) and fathers (8.0%) were most frequently ill. Children (6.0%) and mothers (5.0%) were less affected.

Former tuberculosis patients had a lower body mass index (BMI) at enrolment than HHCs (mean ± SD, 22.9 ± 5.0 vs 25.2 ± 4.8 kg/m^2^; *p* < 0.0001), and 21.2% of tuberculosis patients were underweight. Compared to HHCs, the most prevalent medical risk factors for active disease included alcohol dependency (15.0 vs 5.9%; *p* < 0.0001), glucocorticoid therapy (7.2 vs 2.0%; *p* = 0.004), and diabetes mellitus (7.8 vs 4.0%; *p* = 0.04). Of the diabetic patients, 47% were born outside Germany, mirroring the percentage of foreign-born subjects in the whole cohort (49%). Less prominent risk factors were immunosuppressive treatments, TNF-antagonists therapy, chronic renal failure and HIV infection. IVDU was a strong risk factor for LTBI but was not for PTB.

Fewer patients with PTB were vaccinated with *M. bovis* Bacillus Calmette-Guérin (BCG) than HHCs (44.6 vs 65.0%; *p* value <0.0001).

## Discussion

We identified additional risk factors for LTBI and active pulmonary tuberculosis among HHCs in Germany, a country of low tuberculosis incidence. Our finding that active PTB was reported more frequently in subjects from an urban environment may be seen as a confounding factor. It reflects the demographic structure of German cities, where immigrants in prospering economic regions and cities contribute 9.8–13.4% of the population, compared to 2.8–6.3% in smaller and rural centres [11]. Active PTB was more frequent in males, underweight individuals, patients on glucocorticoid therapy, diabetic patients, and individuals residing in an urban setting. LTBI was most frequently found among family members of patients with PTB, especially when a partner was affected with whom an active sexual relationship existed. LTBI was more prevalent with alcohol and IVDU. In HCWs, only an exposure of more than 20 years was associated with LTBI, while no impact from measures to prevent transmission was found. Of note, there is no standardised protocol for the prevention of transmission in Germany. Most centres use FFP2 respirators for staff and surgical masks for patients. Some staff use a respirator for more than one occasions, some change after every patient contact. Negative pressure isolation is available in very few centres.

Although follow-up was incomplete with 81.6% of enrolled HCWs and 28.4% of HHCs, no contact person developed active tuberculosis within 2 years after exposure, supporting the poor predictive value of IGRA tests found in other trials [8, 12].

In HCWs, only two risk factors for LTBI were identifiable: caring for coughing patients and a longstanding occupational exposure. This is in line with Indian data from a cohort of nursing students. Christopher et al. found that the duration of caring for tuberculosis patients (OR = 1.10; 95% CI 1.04–1.17) and having performed or assisted in sputum collection (OR = 3.57; 95% CI 1.07–11.84) were both significantly associated with TST conversions [13].

In our study, LTBI was found in almost 40% of staff from tuberculosis wards, exceeding the reported frequency in non-specialised nurses in Germany (5.7–8.3%) and France (15.5%) [14, 15] and Portugal (30.5 %) [15]. LTBI remained even more prevalent than in older non-specialised nurses (>55 years) from Germany (25%) or France (33.3%). Only in Portuguese nurses aged ≥45 years, a prevalence of more than 45% was seen, reflecting the higher prevalence of 25/100.000 in Portugal compared to 5–6/100.000 in Germany and France. Our data indicate that in this well-defined, highly exposed population a migration background has no significant impact on the IGRA status and exposure abroad becomes less relevant.

Our study also provides data on LTBI with implications for domestic contact tracing. Of all positive IGRA test results, 55% were found in contacts exposed to a diseased sibling or partner. Sexual activity with the partner increased the probability for LTBI above 50%. Currently, close monitoring of intimate contact persons is recommended but inquisition about a sexual relationship may refine the focus on individuals at risk [10]. In keeping with a recent Norwegian study [16], our analyses support a tracking strategy involving core family members first and farther contact persons second, complementing current algorithms based mainly on proximity and cumulative exposure time [10, 17]. Despite the poor predictive value of a positive IGRA test for the development of active tuberculosis, core contact persons may require a close follow-up [8].

The data also suggest that outside the domestic setting, contacts with substance dependencies warrant thorough investigations due to their increased risk for LTBI. This is in keeping with earlier data comparing IGRA with TST [18]. However, this finding may be population specific, as IVDU appears not to be a risk for LTBI among US prison inmates [19]. Alcohol consumption is associated with both, higher rates of active tuberculosis and medication induced hepatotoxicity—each requiring particular attention after exposure [20].

Surprisingly, IVDU was not associated with active tuberculosis. The correlation of IVDU and LTBI is known from an observational study from Estonia and Latvia and from an observational study in the United States (adjusted OR = 1.34) [21, 22]. Studies suggesting an association between IVDU and active tuberculosis were mostly small, confounded by HIV-infection or lacking control groups [23–28]. According to our data, IVDU is no independent risk factor for the development of active tuberculosis but rather a surrogate marker for precarious social conditions.

This study shows an increased prevalence of tuberculosis in patients with diabetes mellitus in Germany. While there was no correlation in LTBI, the prevalence of diabetes nearly doubled from 4.0% in HHCs to 7.8% in patients with PTB. This phenomenon was found in both foreign-born and Germany born individuals. Patients with PTB were older than HHCs but a multivariate analysis did not detect an age bias. These data require future evaluation. Until now, the epidemiology was considered to be comparable to neighbouring countries like Denmark or Poland, where no association of tuberculosis and diabetes was found [29, 30]. The relationship is not widely acknowledged by German physicians although evidence is accumulating for various other ethnic groups [31–35].

A limitation of our study is the lack of information on preventive chemotherapy in contact persons. A small number of subjects was offered chemotherapy at the time of recruitment, nevertheless no subject developed active tuberculosis. The poor uptake of preventive chemotherapy has been previously described in Germany [9]. It is associated with the de-centralized management of contact persons who are followed up in the health authority offices of 295 German districts.

Another limitation is that the questionnaires were not verified independently. Some data are based on subjective perception. While recruitment of HCWs and HHCs was prospective, recruitment of patients with PTB was prospective (i.e. patients with active tuberculosis or ongoing antimycobacterial therapy) and retrospective (i.e. patients who were cured from tuberculosis at enrolment). The three cohorts were, therefore, independent and not related to each other. This impairs conclusions about cause and effect of PTB on the identified variables. Although participants were recruited from 18 hospitals and 3 health authorities, the data may not be representative for the whole country. However, given the large number of enrolled individuals, the study provides valid information for risk factors of LTBI and active tuberculosis in a Western European country of low tuberculosis incidence.

In conclusion, our study identified risk groups for LTBI and active tuberculosis based on national epidemiologic data as suggested in the latest global strategy plan for tuberculosis prevention by the WHO. We identified male gender, low body weight, glucocorticoid therapy and diabetes mellitus as risk factors for tuberculosis. Risk factors for LTBI included sexual relationship to a diseased household partner and substance abuse. Among HCWs, only long-term professional exposure to tuberculosis patients was related to LTBI. Despite a high frequency of positive IGRA test results among HHCs and HCWs no contact developed active tuberculosis during a 2-year follow-up period.

## Electronic supplementary material

Below is the link to the electronic supplementary material.

Supplementary material 1 (DOC 186 kb)

## Abbreviation

AFB: Acid fast bacilli
BCG: *M. bovis* Bacillus Calmette-Guérin
BMBF: Bundesministerium für Bildung und Forschung
BMI: Body mass index
EEA: European Economic Area
EU: European Union
HCW: Healthcare worker
HHC: Household contact
IGRA: Interferon-gamma release assay
IPT: Isoniazid preventive therapy
IQR: Interquartile range
IVDU: Intravenous drug use
LTBI: Latent tuberculosis infection
OR: Odds ratio
PTB: Pulmonary tuberculosis
SD: Standard deviation
TB: Tuberculosis
TST: Tuberculin skin test
UK: United Kingdom
WHO: World Health Organisation
