# Supplementary material for: Risk for latent and active tuberculosis in Germany
Source: Infection. 2016 Nov 19;45(3):283–90. doi: 10.1007/s15010-016-0963-2 (PMC5488071; doi:10.1007/s15010-016-0963-2)
Supplement: Supplementary file 1 — Supplementary material 1 (DOC 186 kb) [file 15010_2016_963_MOESM1_ESM.doc]

**Online supplement: Table 1 (complete)** Risk factors for LTBI and active TB in household contacts, healthcare workers and patients with pulmonary tuberculosis

|  | | **HHCs** | | | | **HCWs** | | | | **PTBs** | **p-value**  **(all HHCs vs.**  **all HCWs)** | **p-value**  **(all HHCs vs.**  **all**  **PTBs)** | **p-value**  **(all HCWs vs.**  **all**  **PTBs)** |
| --- | --- | --- | --- | --- | --- | --- | --- | --- | --- | --- | --- | --- | --- |
|  |  | **all**  **(n= 522)** | **IGRA -**  **(n= 342)** | **IGRA +**  **(n= 180)** | **p-value**  **IGRA - vs.**  **IGRA + HHCs** | **all**  **(n= 280)** | **IGRA -**  **(n= 171)** | **IGRA +**  **(n= 109)** | **p-value**  **IGRA - vs.**  **IGRA+**  **HCWs** | **all**  **(n= 856)** |  |  |  |
| *Male, n (%)* | | 242/522  (46.4) | 156/342  (45.6) | 86/180  (47.8) | 0.64 | 61/277 (22.0) | 38/169 (22.5) | 23/108  (21.3) | 0.82 | 568/856  (66.4) | **<0.0001** | **<0.0001** | **<0.0001** |
| *Mean (SD) age, years* | | 40.6  (15.0) | 39.1  (14.8) | 43.3  (15.0) | **0.03** | 48.0  (9.3) | 46.3  (9.2) | 50.6  (8.9) | **<0.0001** | 47.1  (16.4) | **<0.0001** | **<0.0001** | 0.38 |
| ***Migration background*** | | | | | | | | | | | | |  |
| *Born in Germany, n (%)* | | 266/519  (53.2) | 191/340  (56.2) | 85/179 (47.5) | 0.06 | 244/276 (88.4) | 151/169 (89.4) | 93/107  (86.9) | 0.54 | 433/855  (50.6) | **<0.0001** | 0.36 | **<0.0001** |
| *Father born in Germany, n (%)* | | 222/514  (43.2) | 156/338  (46.2) | 66/176  (37.5) | 0.06 | 238/276 (86.2) | 145/168 (86.3) | 93/108  (86.1) | 0.96 | 386/849  (45.5) | **<0.0001** | 0.41 | **<0.0001** |
| *Mother born in Germany, n (%)* | | 226/519  (43.6) | 154/340  (45.3) | 72/179  (40.2) | 0.27 | 238/277 (85.9) | 144/169 (85.2) | 94/108  (87.0) | 0.67 | 394/851  (46.3) | **<0.0001** | 0.32 | **<0.0001** |
| ***Domestic risk factors*** | | | | | | | | | | | | | |
| *TB disease of the father, n (%)* | | 50/459 (10.9) | 33/300  (11.0) | 17/159  (10.7) | 0.92 | 13/262 (4.9) | 7/163  (4.3) | 6/99  (6.1) | 0.52 | 61/762  (8.0) | 0.006 | 0.09 | 0.09 |
| *TB disease of the mother, n (%)* | | 34/484 (7.0) | 22/318  (6.9) | 12/166  (7.2) | 0.90 | 16/268 (6.0) | 10/164  (6.1) | 6/104  (5.8) | 0.91 | 39/786  (5.0) | 0.60 | 0.13 | 0.53 |
| *TB disease of the partner, n (%)* | | 61/200 (30.5) | 31/130  (23.9) | 30/70  (42.9) | **0.005** | 5/270 (1.9) | 3/163  (1.8) | 2/107  (1.9) | 1.0 | 58/689  (8.4) | **<0.0001** | **<0.0001** | **0.0003** |
| *TB disease of a grandparent, n (%)* | | 25/289 (8.7) | 16/186  (8.6) | 9/103  (8.7) | 0.97 | 24/204 (11.8) | 12/126  (9.5) | 12/78  (15.4) | 0.21 | 49/540  (9.1) | 0.26 | 0.84 | 0.27 |
| *TB disease of a sibling, n (%)* | | 30/443 (6.8) | 12/294  (4.1) | 18/149  (12.1) | **0.002** | 8/269 (3.0) | 4/162  (2.5) | 4/107  (3.7) | 0.72 | 67/747  (9.0) | **0.03** | 0.16 | **0.01** |
| *TB disease of a child, n (%)* | | 16/136 (11.8) | 8/82  (9.8) | 8/54  (14.8) | 0.37 | 3/266 (1.1) | 2/163  (1.2) | 1/103  (1.0) | 1.0 | 33/549  (6.0) | **<0.0001** | **0.02** | **0.01** |
| ***Environmental risk factors*** | | | | | | | | | | | | | |
| *Current residency,*  *n (%)* | *urban* | 233/254  (91.7) | 153/165  (92.7) | 80/89  (89.9) |  | 101/277  (36.5) | 57/169  (33.7) | 44/108  (40.7) |  | 687/853  (80.5) |  |  |  |
|  | *rural* | 20/254  (7.9) | 12/165  (7.3) | 8/89  (9.0) | 0.35 | 175/277  (63.2) | 111/169 (65.7) | 64/108  (59.3) | 0.39 | 165/853  (19.3) | **<0.0001** | **<0.0001** | **<0.0001** |
|  | *both* | 1/254  (0.4) | 0/165  (0.0) | 1/89  (1.1) |  | 1/277  (0.4) | 1/169  (0.6) | 0/108  (0.0) |  | 1/853  (0.1) |  |  |  |
| *Childhood residency,*  *N (%)* | *urban* | 152/254  (59.8) | 97/165  (58.8) | 55/89  (61.8) |  | 110/276  (39.9) | 55/168  (32.7) | 55/108  (50.9) |  | 481/854  (56.3) |  |  |  |
|  | *rural* | 101/254  (39.8) | 68/165  (41.2) | 33/89  (37.1) | 0.34 | 160/276  (58.0) | 107/168 (63.7) | 53/108  (49.1) | **0.003** | 365/854  (42.7) | **<0.0001** | 0.46 | **<0.0001** |
|  | *both* | 1/254  (0.4) | 0/165  (0.0) | 1/89  (1.1) |  | 6/276  (2.2) | 6/168  (3.6) | 0/108  (0.0) |  | 8/854  (0.9) |  |  |  |
| *Tobacco smoking,*  *n (%)* | *current* | 232/500  (46.4) | 147/326  (45.1) | 85/174  (48.9) |  | 61/260  (23.5) | 38/165  (23.0) | 23/95  (24.2) | 0.83 | 57/116  (49.1) | **<0.0001** | 0.59 | **<0.0001** |
|  | *former* | 313/505 (62.0) | 202/331 (61.0) | 111/174 (63.8) |  | 145/262 (55.3) | 90/166 (54.2) | 55/96 (57.3) | 0.63 | 18/116  (15.2) | 0.08 | **<0.0001** | **<0.0001** |
| ***Medical risk factors*** | | | | | | | | | | | | | |
| *Anti-TNFα treatment, n (%)* | | 0/253  (0.0) | 0/164  (0.0) | 0/89 (0.0) | - | 0/253 (0.0) | 0/162 (0.0) | 0/91 (0.0) | - | 15/843 (1.8) | - | **0.03** | **0.03** |
| *Alcohol dependency, n (%)* | |  | 3/164  (1.8) | 12/89 (13.5) | **<0.0001** |  |  |  |  | 127/846 (15.0) |  | **<0.0001** |  |
| *Chronic renal failure, n (%)* | | 0/252  (0.0) | 0/163  (0.0) | 0/89 (0.0) | - | 5/262 (1.9) | 3/166 (1.8) | 2/96 (2.1) | 1.0 | 13/852 (1.5) | **0.03** | **0.049** | 0.65 |
| *Diabetes mellitus, n (%)* | | 10/251  (4.0) | 4/163  (2.5) | 6/88 (6.8) | 0.09 | 18/260 (6.9) | 14/165 (8.5) | 4/95 (4.2) | 0.22 | 66/852 (7.8) | 0.14 | **0.04** | 0.63 |
| *Glucocorticoids exposure, n (%)* | | 5/251  (2.0) | 3/163 (1.8) | 2/88 (2.3) | 0.82 | 7/254 (2.8) | 5/162 (3.1) | 2/92 (2.2) | 0.67 | 27/374 (7.2) | 0.57 | **0.004** | **0.02** |
| *HIV-positivity, n (%)* | | 0/234  (0.0) | 0/153 (0.0) | 0/81 (0.0) | - | 7/250 (2.8) | 5/160 (3.1) | 2/90 (2.2) | 1.0 | 33/835 (4.0) | **0.01** | **0.002** | 0.38 |
| *Immunosuppressive therapy* | | 1/253  (0.4) | 1/164 (0.6) | 0/89 (0.0) | 0.46 | 8/255 (3.1) | 7/164 (4.3) | 1/91 (1.1) | 0.27 | 28/842 (3.3) | 0.02 | **0.011** | 0.88 |
| *Intravenous drug usage, n (%)* | | 14/253  (5.5) | 2/164  (1.2) | 12/89  (13.5) | **<0.0001** | 5/262  (1.9) | 4/166  (2.4) | 1/96  (1.0) | 0.66 | 29/852  (3.4) | 0.03 | 0.12 | 0.22 |
| *Silicosis, n (%)* | | 0/253  (0.0) | 0/164  (0.0) | 0/89  (0.0) | - | 2/261  (0.8) | 1/166  (0.6) | 1/95  (1.1) | 1.0 | 3/849  (0.4) | 0.16 | 0.34 | 0.42 |
| *Gastrectomy, n (%)* | | 3/252  (1.2) | 2/163  (1.2) | 1/89  (1.1) | 0.94 | 1/261  (0.4) | 0/166  (0.0) | 1/95  (1.1) | 0.19 | 12/854  (1.4) | 0.30 | 0.80 | 0.19 |
| *Jejuno-ileal by-pass, n (%)* | |  | 0/163  (0.0) | 0/88  (0.0) | - |  |  |  |  | 4/851  (0.5) |  | 0.28 |  |
| *Malignant disease, n (%)* | | 3/252  (1.2) | 1/164  (0.6) | 2/88  (2.3) | 0.25 | 5/257  (2.0) | 3/163  (1.8) | 2/94  (2.1) | 1.0 | 29/853  (3.4) | 0.49 | 0.07 | 0.26 |
| *Organ transplantation, n (%)* | | 0/253  (0.0) | 0/164  (0.0) | 0/89  (0.0) | - | 2/259  (0.8) | 2/165  (1.2) | 0/94  (0.0) | 0.54 | 1/854  (0.1) | 0.16 | 0.59 | 0.05 |
| ***Previous mycobacterial exposure and diagnostics*** | | | | | | | | | | | | | |
| *BCG vaccination, n (%)* | | 193/297  (65.0) | 122/187  (65.2) | 71/110  (64.6) | 0.90 | 147/251  (58.6) | 88/155  (56.8) | 59/96  (61.5) | 0.46 | 193/433  (44.6) | 0.12 | **<0.0001** | **0.0004** |
| *Previous active TB, n (%)* | |  | 1/336  (0.3) | 8/179  (4.5) | **0.001** |  | 11/168  (6.6) | 10/104  (9.6) | 0.36 |  |  |  |  |
| *Preventive treatment post exposure, n (%)* | |  | 3/109  (2.8) | 11/59  (18.6) | **<0.0001** |  |  |  |  |  |  |  |  |
| *Previous TST performed, n (%)* | |  | 74/292  (25.3) | 52/150  (34.7) | 0.04 |  | 156/167 (93.4) | 103/106 (97.2) | 0.17 |  |  |  |  |
| *Previous TST result, n (%)* | *Positive* |  | 20/66  (30.3) | 18/47  (38.3) | 0.49 |  |  |  |  |  |  |  |  |
|  | *Negative* |  | 45/66  (68.2) | 29/47  (61.7) |  |  |  |  |  |  |  |  |  |
|  | *Negative and positive* |  | 1/66  (1.5) | 0/47  (0.0) |  |  |  |  |  |  |  |  |  |
| *Previous IGRA test performed*  *n (%)* | | 26/502  (5.2) | 12/329  (3.7) | 14/173  (8.1) | **0.03** | 40/265  (15.1) | 19/161  (11.8) | 21/104  (20.2) | 0.06 |  | **<0.0001** |  |  |
| *Previous positive IGRA test result,*  *n (%)* | | 6/26  (23.1) | 1/12  (8.3) | 5/14  (35.7) | 0.10 | 21/41  (51.2) | 5/22  (22.7) | 16/19  (84.2) | **<0.0001** |  | **0.02** |  |  |

**Online supplementTable 2** Specific risk factors for LTBI in healthcare workers

|  | | **IGRA-Negative**  **(n= 171)** | **IGRA-Positive**  **(n= 109)** | **p-value** |
| --- | --- | --- | --- | --- |
| **Professional exposure** | | | | |
| Duration of professional TB exposure  n (%) | <5 years | 28/169 (16.6) | 15/104 (14.4) | **0.004** |
|  | <10 years | 37/169 (21.9) | 12/104 (11.5) |  |
|  | 10-20 years | 62/169 (36.7) | 30/104 (28.9) |  |
|  | >20 years | 42/169 (24.9) | 47/104 (45.2) |  |
| Number of TB patients managed,  annual median in 5 years (IQR) | | 60 (40-100) | 80 (40-120) | 0.23 |
| Exposure to coughing TB patients  n (%) | Never | 17/130 (13.1) | 25/91 (27.5) | **0.02** |
|  | Occasionally | 52/130 (40.0) | 26/91 (28.6) |  |
|  | Frequently | 61/130 (46.9) | 40/91 (44.0) |  |
| Profession, n (%) | Nurse | 118/169 (69.8) | 71/107 (76.4) | 0.11 |
|  | Doctor | 31/169 (18.3) | 14/107 (13.1) |  |
|  | Other | 20/169 (11.8) | 22/107 (20.6) |  |
| Unprotected exposure to TB patients  n (%) | Never | 35/128 (27.3) | 23/87 (26.4) | 0.13 |
|  | Occasionally | 78/128 (60.9) | 45/87 (51.7) |  |
|  | Frequently | 15/128 (11.7) | 19/87 (21.8) |  |
| Management of mechanically ventilated TB patients, n (%) | Never | 88/129 (68.2) | 64/89 (71.9) | 0.56 |
|  | Occasionally | 41/129 (31.8) | 25/89 (28.1) |  |
| Cardiopulmonary resuscitation in TB patients, n (%) | Never | 81/127 (63.8) | 61/87 (70.1) | 0.34 |
|  | Occasionally | 46/127 (36.2) | 26/87 (29.9) |  |
| Endoscopic procedures in TB patients,  n (%) | Never | 82/128 (64.1) | 47/87 (54.0) | 0.31 |
|  | Occasionally | 35/128 (27.3) | 29/87 (33.3) |  |
|  | Frequently | 11/128 (8.6) | 11/87 (12.6) |  |
| **Protective measurements** | | | | |
| Use of surgical face mask, n (%) | | 105/139 (75.5) | 56/81 (69.1) | 0.30 |
| Use of FFP-2 respirators, n (%) | | 92/141 (65.3) | 59/92 (64.1) | 0.86 |
| Use of whitecoats, n (%) | | 126/162 (77.8) | 75/101 (74.3) | 0.51 |
| Use of clean room ventilation, n (%) | | 35/143 (24.5) | 16/84 (19.1) | 0.34 |
| **Occupational health diagnostics** | | | | |
| Previous TST, n (%) | | 156/167 (93.4) | 103/106 (97.2) | 0.17 |
| Previous positive TST result, n (%) | | 72/135 (53.3) | 77/89 (86.5) | **<0.0001** |
| Previous IGRA, n (%) | | 19/161 (11.8) | 21/104 (20.2) | 0.06 |
| Previous positive IGRA, n (%) | | 5/22 (22.7) | 16/19 (84.2) | **<0.0001** |
| IGRA system, n (%) | QFT | 104/171 (60.8) | 60/109 (55.1) | 0.34 |
|  | TSPOT-TB | 67/171 (39.2) | 49/109 (45.0) |  |

SD: Standard Deviation; BCG: Bacillus Calmette-Guerin; TB: Tuberculosis; IGRA: Interferon-γ Release Assay; QFT: Quantiferon Test; TST: Tuberculin Skin Testing; IQR: InterQuartile Range

**Online supplement** **Table 3. Logistic regression analysis assessing the relationship between IGRA positivity and clinical, epidemiological, and demographic variables household contacts and healthcare workers.**

|  | **Household contacts** | | | | **Healthcare workers** | | | |
| --- | --- | --- | --- | --- | --- | --- | --- | --- |
|  | **Univariate** | | **Multivariate** | | **Univariate** | | **Multivariate** | |
|  | **OR (95%CI)** | **p-value** | **OR (95%CI)** | **p-value** | **OR (95%CI)** | **p-value** | **OR (95%CI)** | **p-value** |
| Male | 1.09 (0.76-1.57) | 0.64 | - | - | 0.93 (0.52-1.68) | 0.82 | - | - |
| Age, years | 1.02 (1.00-1.04) | 0.03 | 0.99  (0.97-1.02) | 0.72 | 1.06 (1.03-1.09) | <0.0001 | 1.08 (1.04-1.13) | <0.0001 |
| Born in Germany | 0.71 (0.49-1.01) | 0.06 | - | - | 0.79 (0.38-1.67) | 0.54 | - | - |
| Father born in Germany | 0.7 (0.48-1.02) | 0.06 | - | - | 0.98 (0.49-1.98) | 0.96 | - | - |
| Mother born in Germany | 0.81 (0.56-1.17) | 0.27 | - | - | 1.17 (0.58-2.36) | 0.67 | - | - |
| TB disease of the father | 0.97 (0.52-1.80) | 0.92 | - | - | 1.44 (0.47-4.41) | 0.53 | - | - |
| TB disease of the mother | 1.05 (0.51-2.18) | 0.90 | - | - | 0.94 (0.33-2.68) | 0.91 | - | - |
| TB disease of the partner | 2.40 (1.29-4.46) | 0.006 | 4.39  (1.88-10.26) | 0.001 | 1.02 (0.17-6.18) | 0.99 | - | - |
| TB disease of a grandparent | 1.02 (0.43-2.39) | 0.97 | - | - | 1.73 (0.73-4.06) | 0.21 | - | - |
| TB disease of a sibling | 3.23 (1.51-6.90) | 0.002 | 1.23  (0.28-5.36) | 0.79 | 1.53 (0.38-6.27) | 0.55 | - | - |
| TB disease of a child | 1.61 (0.56-4.58) | 0.37 | - | - | 0.79 (0.07-8.82) | 0.85 | - | - |
| Tobacco smoking | 1.16 (0.80-1.68) | 0.42 | - | - | 1.07 (0.59-1.93) | 0.83 | - | - |
| Anti-TNFa treatment | - | - | - | - | - | - | - | - |
| Chronic renal failure | - | - | - | - | 1.16 (0.19-7.04) | 0.88 | - | - |
| Diabetes mellitus | 2.91 (0.80-10.60) | 0.11 | - | - | 0.47 (0.15-1.48) | 0.20 | - | - |
| Glucocorticoids exposure | 1.24 (0.20-7.57) | 0.82 | - | - | 0.70 (0.13-3.67) | 0.67 | - | - |
| HIV-positivity | - | - | - | - | 0.71 (0.13-3.71) | 0.68 | - | - |
| Immunosuppressive therapy | - | - | - | - | 0.25 (0.03-2.06) | 0.20 | - | - |
| Intravenous drug usage | 12.62 (2.76-57.79) | 0.001 | - | -* | 0.43 (0.05-3.87) | 0.45 | - | - |
| Silicosis | - | - | - | - | 1.76 (0.11-28.39) | 0.69 | - | - |
| Gastrectomy | 0.92 (0.81-10.23) | 0.94 | - | - | - | - | - | - |
| Malignant disease | 3.79 (0.34-42.40) | 0.28 | - | - | 1.16 (0.19-7.07) | 0.87 | - | - |
| Organ transplantation | - | - | - | - | - | - | - | - |
| BCG vaccination | 0.97 (0.59-1.59) | 0.90 | - | - | 1.21 (0.72-2.04) | 0.46 | - | - |
| Previous active TB | 15.67 (1.94-126.33) | 0.01 | - | -* | 1.52 (0.62-3.71) | 0.36 | - | - |
| Childhood urban residency | 1.28 (0.77-2.12) | 0.34 | - | - | 2.13 (1.30-3.50) | 0.003 | 2.14 (1.17-3.92) | 0.01 |
| Exposure to coughing TB patients | - | - | - | - | 0.60 (0.41-0.88) | 0.009 | 0.72 () | 0.13 |
| Duration of professional TB exposure | - | - | - | - | 1.39 (1.09-1.77) | 0.008 | 1.03 (0.75-1.41) | 0.89 |

CI: confidence interval; OR: odds ratio

*Dropped from the final multivariate model because they predict success perfectly
